# The impact of caregiver mental health on child prosocial behavior: A longitudinal analysis of children and caregivers in KwaZulu-Natal, South Africa

**DOI:** 10.1371/journal.pone.0290788

**Published:** 2023-10-20

**Authors:** Gabriella A. Norwitz, Chris Desmond, Rachel S. Gruver, Jane D. Kvalsvig, Amaleah F. Mirti, Shuaib Kauchali, Leslie L. Davidson

**Affiliations:** 1 Department of Epidemiology, Mailman School of Public Health, Columbia University, New York, NY, United States of America; 2 Center for Rural Health, University of KwaZulu-Natal, KwaZulu-Natal, South Africa; 3 Department of Public Health, University of KwaZulu-Natal, KwaZulu-Natal, South Africa; 4 Maternal, Adolescent, and Child Health Institute NPC (MatCH), Durban, South Africa; 5 Department of Pediatrics, Columbia College of Physicians and Surgeons, New York, NY, United States of America; University of Rome La Sapienza: Universita degli Studi di Roma La Sapienza, ITALY

## Abstract

**Background:**

Prosocial behavior has positive social, cognitive, and physical health effects on the individual exhibiting the behavior as well as on society as a whole, and is integral to overall mental and physical wellbeing. The development of prosocial behavior is rooted in early childhood and learned through observation. As such, those spending time with children, especially their caregiver, play a critical role in their prosocial development. The current study investigates the impact of caregiver mental health on the prosocial development of young children over time.

**Methods:**

This paper presents a secondary analysis of child prosocial development in the Asenze Study, a longitudinal, population-based cohort study based in KwaZulu-Natal, South Africa. Children were followed-up over time from an average age of five to seven years along with their caregivers. Linear GEE regression analysis was used to assess whether a change in presence of a mental health disorder in a caregiver during this 2-year interval (using the Client Diagnostic Questionnaire) impacted the development of their child’s prosocial behavior (using the Strengths and Difficulties Questionnaire).

**Results:**

After adjusting for early child-care, child HIV status, SDQ child prosocial subscale, SDQ total difficulties score, and household order score (CHAOS), children whose caregivers acquired a mental health disorder had a significantly smaller increase in prosocial behavioral development compared to children whose caregivers never had a mental health disorder.

**Conclusions:**

Identifying contextually relevant modifiable factors such as this will help stimulate the development of interventions to promote prosocial development in childhood.

## Introduction

Prosocial behavior refers to behavior through which an individual benefits others. It includes such actions as cooperation, collaboration, sharing, helpfulness, donation, volunteering, and empathy [1, 2]. Individuals who exhibit high levels of prosocial behavior, in general, have better overall mental wellbeing [3] as well as better social, cognitive, and physical health outcomes. This includes more positive peer relationships [4–6], increased social acceptance [4, 5, 7], higher academic achievements [4, 7, 8], increased happiness and positive mood [5, 9, 10], higher overall life satisfaction [10], fewer behavioral problems, aggression, and stress [6, 7, 10], lower rates of disease, and greater longevity [10]. Prosocial behavior develops in early childhood through observational learning and reaches a plateau in adolescence, suggesting that early childhood is a critical time period for prosocial development [10, 11]. The people who surround and raise a child, especially the primary caregiver, play a pivotal role in prosocial development through modeling prosocial behavior and positively reinforcing the child’s use of such behavior [7, 12]. In this way, caregivers guide the development of prosocial behavior in their children, laying the foundation for their social interactions later in life. One study found that children whose mothers had a mental health diagnosis of depression or anorexia nervosa “… showed less cooperation and coherence during the dialogues about their emotions compared to the children of the mothers in the non-clinical group” [13]. By impacting the emotional dialogue between a caregiver and child, it is likely that a caregiver’s mental health condition may adversely impact a child’s prosocial behavior development.

Previous literature has shown an association between caregiver mental health and child prosocial development; however, results were inconsistent in terms of the change in childhood prosocial behavior over time, their major drivers, and the impact of confounders [14]. Additionally, few studies have analyzed prosocial behavioral longitudinally, and, to our knowledge, none have examined this association in a sample in which not all caregivers are mothers [5, 11, 15]. Few childhood behavior screens measure prosocial behavior, (Child Behavior Checklist [16], Achenbach [17], Conners [18], and Ages and Stages Questionnaire [19]) and even when they do, for example the Strengths and Difficulties Questionnaire (SDQ), the prosocial behavior subscale is often not analyzed as it is conceptually different from the presence of behavioral difficulties, which is assessed using the other four SDQ subscales [20, 21]. For example, a longitudinal study of a Zimbabwean cohort of maternal caregiver-child dyads found that exposure to a caregiver mental disorder adversely impacted vocabulary skill acquisition in the child [22].

The objective of the current study builds on previous literature exploring how caregiver mental health affects child development. Using data from the Asenze Cohort, we conducted a longitudinal study of caregivers and children in KwaZulu-Natal, South Africa, to investigate the impact of caregiver mental health on prosocial development of children over time, and analyzed the effect of potential confounders. South Africa is a particularly appropriate setting for such a study as its history of apartheid and delayed governmental action against HIV has resulted in one of the highest rates of persons living with HIV/AIDS [23], poverty, social inequality [24], and psychiatric disorders anywhere in the world.

## Materials and methods

### Ethics statement

The Asenze Cohort Study received ethical approval for all waves of the study and any modifications from the Biomedical Research Ethics Committee (BREC # BF036/07) of the University of KwaZulu-Natal and from the Institutional Review Board of Columbia University (IRB No. AAAC2559). Initial support was also received from local authority councils and from the local district health committee and the local district Board of Education. Written informed consent was obtained from primary caregivers on behalf of themselves and their child before enrollment.

### Data source and sample selection

This analysis uses data from the first two waves of the Asenze Study, a longitudinal population-based prospective cohort study of children, on average five years old at wave 1 and seven years old at wave 2, and their caregivers in KwaZulu-Natal, South Africa. KwaZulu-Natal is the second most populous South African province representing 20% of the national population, 11.5 million people [25, 26].

The Asenze Cohort Study began in 2008 with a door-to-door survey of five contiguous local authority tribal areas in peri-urban areas of the Valley of a Thousand Hills in Durban, South Africa. The study offices were based out of The Valley Trust, a leading South African non-governmental organization. The population in the Valley of a Thousand Hills is comprised primarily of Zulu people and all participants were African isiZulu-speaking. All children between 4 and 6 years old were eligible to participate along with their primary caregivers. Four to 6 years old was selected as it is an age of emerging neurodisability and allows for the study of children before and after they enter primary school. The primary caregiver was defined as the person mainly responsible for the care of and decision making for the child. There was high enrollment with 87% of those eligible completing wave 1 and almost 90% retention between waves 1 and 2. Study protocol details have been previously published [27]. Wave 1 (2008–2010) and wave 2 (2010–2014) aimed to assess child physical, cognitive, and social growth as well as factors that may impact this growth, such as caregiver characteristics and socioeconomic environments. Due to the ongoing burden of health, social, and economic inequities that exist in KwaZulu-Natal, the data collected during the course of this study have the potential to provide important insights to better understand early interpersonal factors that affect child development and develop strategies that could aid in the positive development of children and ultimately their functioning as adolescents and adults.

### Client diagnostic questionnaire (CDQ)

#### CDQ validation and administration

The CDQ was designed in 2004 to screen for a range of mental health disorders in populations highly prevalent for HIV infection [28]. It is intended to be administered by non-mental health professionals, thereby facilitating use in low resource settings that have few mental health providers such as KwaZulu-Natal. The CDQ aligns with diagnostic criteria in the Diagnostic and Statistical Manual of Mental Disorders-IV (DSM-IV) and is based on the PRIME-MD (a mental health tool developed for use in the primary care setting) with the addition of a post-traumatic stress disorder (PTSD) module due to the high level of psychological trauma seen in HIV-positive populations. Although “disorder” is used, the CDQ is a screening tool and not a clinical assessment resulting in a definitive diagnosis.

For the Asenze Cohort Study, the CDQ was translated into isiZulu and back-translated by bilingual isiZulu and English South African assessors. The CDQ was administered to primary caregivers by assessors who, though experienced in assessing child development and behavior, were trained and supervised by a South African psychologist, as they did not have previous training in adult mental health disorders. A CDQ validation study was performed on a convenience sample of 322 primary caregivers from Asenze wave 2. Their demographic characteristics reflected those of the main study cohort. These primary caregivers were first administered the CDQ by isiZulu-speaking assessors and then the same day by an isiZulu-speaking South African clinical psychologist who was blinded to the original CDQ results. This internal validation reported the CDQ screen had high sensitivity (74%), specificity (79%), and overall accuracy (79%) when used to assess ‘any psychiatric disorder’ as well as high construct validity when compared with a more widely used measure of mental health, the Short Form 36 Health Questionnaire (SF-36) [29, 30].

### CDQ operationalization

At both waves 1 and 2, primary caregivers were interviewed using a structured CDQ covering five mental health disorders (major depressive disorder, other depressive disorder, panic disorder, generalized anxiety disorder, and/or PTSD). For this analysis, at each time point, caregivers were categorized as either (1) having one or more mental health disorders, or (2) absence of all disorders. Then, they were placed into four categories describing longitudinal patterns of mental health disorder: (1) persistent presence of a mental health disorder (mental health disorder present at both waves), (2) persistent absence of a mental health disorder (no mental health disorder present at either wave), (3) acquisition of a mental health disorder (a mental health disorder at wave 2 but not 1), and (4) resolution of a mental health disorder (a mental health disorder at wave 1 but not 2).

### Strengths and difficulties questionnaire (SDQ)

#### SDQ validation and administration

The SDQ is a brief, open-access questionnaire screening child behavior (ages 3 to 17 years) [20, 21]. The parental version of the SDQ contains 25 questions broken into five subscales covering emotional symptoms, conduct problems, hyperactivity/inattention, peer relationship problems, and prosocial behaviors [20]. The first four subscales are summed and referred to as the total difficulties score, which assesses behavioral problems. The fifth addresses prosocial behavior. The SDQ can be administered by lay counselors making it convenient for community settings. The SDQ has been translated into 89 dialects and languages, and shows high reliability and validity across a variety of cultures and languages [21]. It also is highly correlated with older measures of child behavior, such as the Rutter Questionnaire [20] and the Child Behavior Checklist [31].

The SDQ was translated into isiZulu and back-translated, a process that also involved focus groups with South African isiZulu speakers and a linguist. It was then reviewed by one of the SDQ creators and subsequently accepted by the SDQ website as the official isiZulu version of the SDQ [21]. The SDQ was administered to primary caregivers by lay assessors who were trained and supervised by a South African child psychologist. A SDQ validation study was performed in the Asenze Cohort Study population using the entire sample of children at both waves 1 and 2. This validation reported a marginally acceptable internal consistency for the SDQ prosocial subscale (Cronbach’s alpha of 0.57 for wave 1 and 2) [29].

#### SDQ operationalization

The prosocial subscale of the SDQ is of particular interest for this analysis. The prosocial behavior subscale included five statements asking primary caregivers to report on their child’s behavior over the last six months: (1) ‘considerate of other people’s feelings,’ (2) ‘shares readily with other children (treats, toys, pencils etc.),’ (3) ‘helpful if someone is hurt, upset or feeling ill,’ (4) ‘kind to younger children,’ and (5) ‘often volunteers to help others (parents, teachers, other children).’ These statements were scored on a three-point Likert scale: 0 = ‘not true,’ 1 = ‘somewhat true,’ 2 = ‘certainly true.’ A single composite prosocial score was then generated.

The subscales of the SDQ are often treated continuously [11, 15, 32, 33] although some studies report ranking due to non-normative distributions [33–36]. The prosocial subscale scores for both wave 1 and 2 were left skewed. We therefore categorized the prosocial scores using a technique from a community-based sample in the United Kingdom classifying prosocial scores into four categories: very low (score 0–5), low (score 6), slightly lowered (score 7), and high (score 8–10), which was designed to capture 80% of the study population in the ‘high’ category [21]. For our study sample, 80–90% of our sample were captured in the ‘high’ category for wave 1 and 2. This categorization was used for the cross-sectional analysis at wave 1 and 2. For the longitudinal analysis, we went back to the original continuous prosocial scores and calculated a ‘change in child prosocial score’ by subtracting each child’s wave 1 from their wave 2 prosocial score, which resulted in a normally distributed continuous outcome variable and then adjusted for their baseline score in the analysis.

### Covariates (assessed at wave 1)

The child’s age and sex were reported by the primary caregiver. Early child-care was coded as ‘yes’ (if the caregiver reported the child attending either crèche, out of home care, or preschool, rather than at home care) or ‘no’. Child age [37], sex [3, 33, 34], and early child-care [38] were included as they have been reported to affect prosocial behavior. Children were given rapid HIV tests at wave 1 to assess their HIV status. However, if a test was not administered and the child was tested prior to the study assessment, then their HIV status was assigned as reported by the primary caregiver. Child HIV status was included because of both its direct and indirect impacts on a child’s social, cognitive, and physical development [26, 39, 40]. Child SDQ baseline prosocial score was included because the impact of primary caregiver mental health disorders on the prosocial scale may vary by a child’s initial prosocial score. Child SDQ baseline total difficulties score was included to capture the presence of a behavioral problem [11, 15, 32, 33, 41] as literature suggests that behavioral problems may impact the ability of children to exhibit prosocial behaviors [3, 42]. The Confusion, Hubbub, and Order Scale (CHAOS) captured confusion and disorganization within a child’s home and is measured as an additive total of 12 individual questions.

The primary caregiver’s age and sex were self-reported. Primary sex was included as mothers and fathers have been shown to differentially contribute to child prosocial behavioral development [7, 35]. Although some primary caregivers were fathers, the numbers were small. As such, there was inadequate power to create sub-analyses of caregiver influence by sex. Primary caregiver HIV status was determined primarily by the results of a rapid HIV test given at wave 1 assessment; if not tested at that time, their HIV status was based on self-reporting. Primary caregiver HIV status was included as it may negatively impact a primary caregiver’s ability to care for their child and also because it is known to increase the risk of acquiring a mental health disorder [30]. Primary caregivers were also asked a series of questions about their experience with intimate partner violence (IPV) adapted from the South African Medical Research Council [43] and were categorized as ‘ever experienced IPV’ or not. A primary caregiver having ever experienced IPV was included as it is known to have an impact on the health [36, 37] and behavioral development of children, as noted in an earlier Asenze Cohort Study publication [36, 37]. The WHO AUDIT Screen is composed of 10 questions that sum to up to 40 [44–46]. Positive primary caregiver ‘hazardous alcohol consumption’ was noted for primary caregivers who scored ≥8 on the Alcohol Use Disorders Identification Test (AUDIT), the accepted standard cut off for this measure [47]. Early Asenze Cohort Study publications note the potential effects of alcohol use on primary caregiver mental health [30] and child behavior [48, 49]. A tertile wealth index was created for the Asenze Cohort Study [50] using a principle components approach [51] based on household assets [52]. Wealth index was included as a covariate as published literature suggests that economic deprivation is associated with both adverse mental health and suboptimal psychological health and development in children [15].

### Statistical analysis

At wave 1 and 2, we examined the association between primary caregiver mental health disorder and child prosocial subscale score (treated as a four-level categorical variable) using multinomial logistic generalized estimating equation (GEE) regression models with robust standard error to account for correlations between children that shared a primary caregiver.

We then computed the unadjusted and adjusted association between change in presence of a primary caregiver mental health disorder (using persistent absence as a reference category) and change in child prosocial score over time (treated as a continuous variable). We used a linear GEE regression model. Twelve covariates from wave 1 were assessed for their association with change in presence of a primary caregiver mental health disorder using multinomial logistic GEE regression models, for their association with change in the child prosocial score using linear GEE regression models, and to see if their individual inclusion changed the unadjusted parameter estimate using univariate linear GEE models. Only covariates that were (1) significantly related to change in presence of a primary caregiver mental health disorder and change in the child prosocial score at 90% level of significance (*α*<0.10) and (2) changed the parameter estimate by at least 10% in one of the primary caregiver mental health categories were included in the final adjusted analysis, resulting in five included covariates. These five variables (early child-care, child HIV status, child prosocial score, child SDQ total difficulties score, and CHAOS scores) were then adjusted for in the final analysis. A 95% significance level (*α*<0.05) was used for all analytical conclusions (indicated by *). All analyses were performed using SAS software version 9.4.

## Results

### Study population

**Fig 1:**
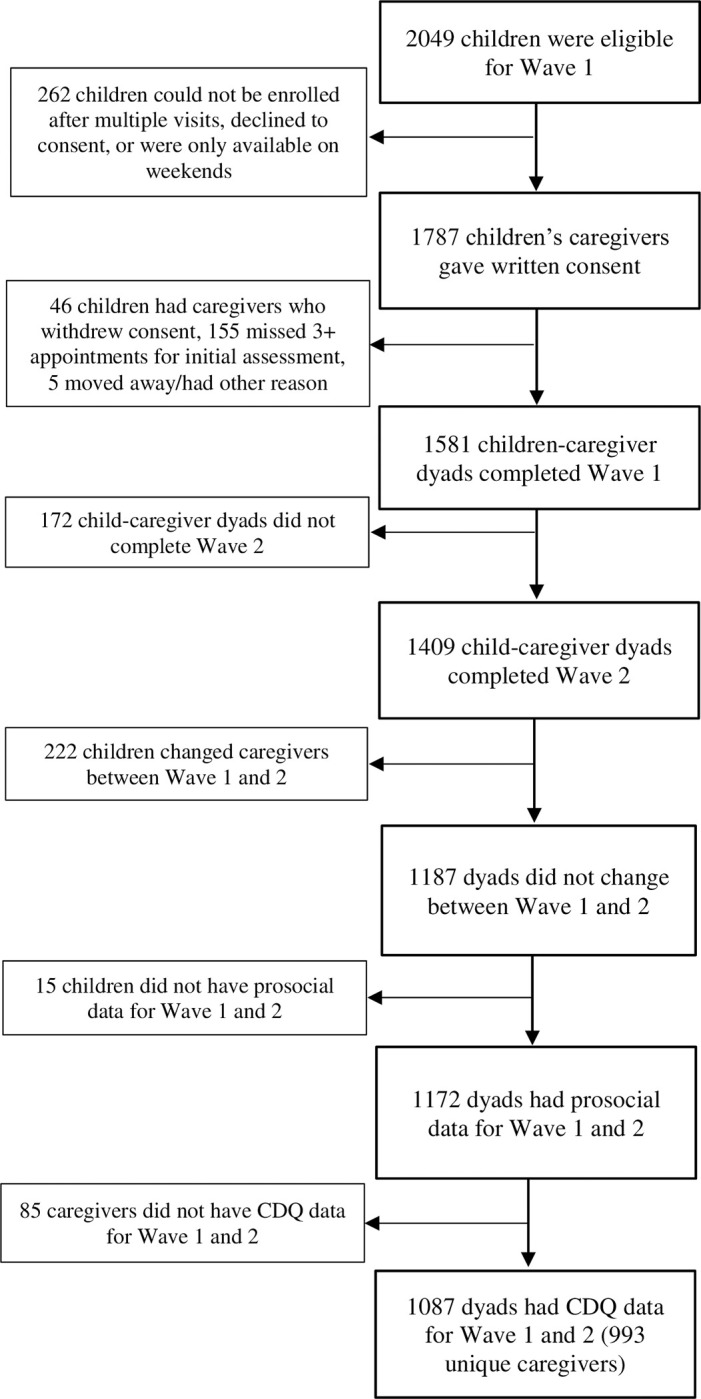
Flow diagram of study population.

For this analysis, certain children and their caregivers were excluded for reasons enumerated in Fig 1. Of note, children who changed caregivers between waves 1 and 2 were excluded (Fig 1). The characteristics of the remaining 1087 children and 993 unique primary caregivers in waves 1 and 2 included in this analysis are shown in Table 1, including demographic information, physical and psychological health, and health behaviors. Of note, 904 primary caregivers had one child in the study, 84 primary caregivers had two children, and five primary caregivers had three children. 70% of primary caregivers in this sample were mothers and less than 1% were fathers. Primary caregivers had high rates of HIV (25.7% in wave 1 and 32.1% in wave 2) and experience with IPV (44.4% in wave 1 and 50.8% in wave 2).

**Table 1:** Characteristics of the study population.

			Wave 1	Wave 2
**Children (N = 1087)**				
Age in years, mean (SD)			4.96 (0.59)	6.94 (0.59)
Female Sex, N (%)			540 (49.7%)	540 (49.7%)
Early child-care (crèche or preschool), Yes N (%)			679 (62.6%) Missing = 3	Not collected
HIV status, N (%)	HIV+		44 (4.0%)	49 (4.5%)
	HIV-		889 (81.8%)	984 (90.5%)
	Unknown		154 (14.2%)	54 (5.0%)
SDQ prosocial behavior score (treated as a categorical variable), N (%)	Very low (score = 0–5)		68 (6.2%)	45 (4.1%)
	Low (score = 6)		63 (5.8%)	38 (3.5%)
	Slightly lower (score = 7)		76 (7.0%)	37 (3.4%)
	High (score = 8–10)		880 (81.0%)	967 (89.0%)
SDQ prosocial behavior score (treated as a continuous variable), mean (SD), max score 10			8.50 (1.82)	9.07 (1.60)
SDQ total difficulties score, mean (SD), max score 40			15.75 (6.38)	13.93 (6.42)
Confusion, Hubbub, and Order Scale Score (CHAOS), mean (SD), max score 12			6.62 (1.70)	6.76 (1.66) Missing = 3
**Primary caregivers (N = 993)**				
Age in years, mean (SD)			37.31 (13.72) Missing = 235	38.32 (13.02)
Female **Sex**, N (%)			964 (98.8%) Missing = 17	973 (98.0%)
Primary caregiver type, N (%)	Mother		697 (70.2%)	702 (70.7%)
	Father		8 (0.8%)	8 (0.8%)
	Grandmother		195 (19.6%)	196 (19.7%)
	Other		93 (9.4%)	87 (8.8%)
Highest education completed, N (%)	None		52 (10.8%)	Not collected
	Grade 1–7 (primary)		111 (23.0%)	
	Grade 8–11 (high school)		213 (44.1%)	
	Grade 12 (matric)		89 (18.4%)	
	> Grade 12 (college/tertiary education)		18 (3.7%)	
	Missing		510	
Mental health disorders†, Yes N (%)	Any mental health disorder†		313 (31.5%)	218 (22.0%)
	Major depressive disorder†		50 (5.0%)	46 (4.7%)
	Other depressive disorder†		40 (4.0%) Missing = 1	21 (2.1%) Missing = 1
	Panic disorder†		63 (6.3%)	53 (5.3%) Missing = 1
	Generalized anxiety disorder†		57 (5.7%)	48 (4.9%) Missing = 3
	Post-traumatic stress disorder†		248 (25.0%)	148 (15.1%) Missing = 14
HIV status, N (%)	HIV+		255 (25.7%)	319 (32.1%)
	HIV-		650 (65.4%)	641 (64.6%)
	Unknown		88 (8.9%)	33 (3.3%)
Ever experienced intimate partner violence (IPV), Yes N (%)			440 (44.4%) Missing = 3	458 (50.8%) Missing = 92
Hazardous alcohol consumption (AUDIT score ≥8), Yes N (%)			31 (3.1%) Missing = 1	52 (5.3%) Missing = 6

**†**Although “disorder” is used, the CDQ is a screening tool and not a clinical assessment resulting in a definitive diagnosis

“Missing” was only included for variables with missing data.

### Primary caregiver mental health disorder and child prosocial behavior scores at wave 1 and 2

In wave 1 and 2 respectively, 31.5% and 22.0% of primary caregivers had one or more mental health disorders (with PTSD being the most common single mental health disorder identified), and 81.0% and 89.0% of children were categorized as having high scores on the prosocial behavior subscale (Table 1). In unadjusted analyses, children whose primary caregiver had a mental health disorder had significantly higher prosocial scores compared to those children with caregivers without a mental health disorder at both wave 1 (OR:1.38, 95% CI: 1.01, 1.89; p = 0.0432) and wave 2 (OR: 1.73, 95% CI: 1.13, 2.65; p = 0.012) (Table 2). However, after testing multiple covariates and adjusting for child SDQ total difficulties score in wave 1, this association was no longer significant (p = 0.3369) (Table 2).

**Table 2:** Association between primary caregiver mental health disorder† and child prosocial behavior scores at wave 1 and 2.

	*β*	95% CI		p-value	OR	OR 95% CI	
Wave 1 (Unadjusted)	0.32	0.01	0.64	0.0432*	1.38	1.01	1.89
Wave 1 (Adjusted**‡**)	0.16	-0.17	0.50	0.3369	1.18	0.84	1.64
Wave 2	0.55	0.12	0.97	0.0120*	1.73	1.13	2.65

Multinomial logistic GEE regression

**†**Although “disorder” is used, the CDQ is a screening tool and not a clinical assessment resulting in a definitive diagnosis

**‡**Adjusting for child SDQ total difficulties score

### Change in presence of a primary caregiver mental health disorder and change in child prosocial behavior score between wave 1 and 2

Between wave 1 and 2, the majority (56.1%) of primary caregivers did not have a mental health disorder at either wave (persistent absence), 21.3% had a mental health disorder that was resolved by wave 2 (resolution), 12.0% had a mental health disorder that was acquired by wave 2 (acquisition), and 10.6% had a mental health disorder at both wave 1 and 2 (persistent presence) (Table 3). The average change in child prosocial score between wave 1 and 2 was 0.57 with a standard deviation of 1.95. When stratified, children with primary caregivers who had resolved mental health disorders had the greatest change in prosocial score (0.67), followed by persistent absence (0.59), acquisition (0.49), and persistent presence (0.41) (Table 3).

**Table 3:** Change in presence of a primary caregiver mental health disorder† and change in child prosocial behavior score between wave 1 and 2.

			Change in Child Prosocial Score, mean (SD)
Overall Change in Child Prosocial Score			0.57 (1.95)
**Change in Presence of a Primary caregiver Mental Health Disorder†, N (%)**	Persistent absence	610 (56.1%)	0.59 (1.80)
	Persistent presence	115 (10.6%)	0.41 (2.00)
	Resolution	232 (21.3%)	0.67 (2.10)
	Acquisition	130 (12.0%)	0.49 (2.27)

**†**Although “disorder” is used, the CDQ is a screening tool and not a clinical assessment resulting in a definitive diagnosis

In the unadjusted analysis, change in presence of a primary caregiver mental health disorder was not associated with change in a child’s prosocial score from wave 1 to wave 2, although the observed pattern was directionally consistent with our hypothesis (Table 4). After adjusting for early child-care, child HIV status, child SDQ baseline prosocial score, child SDQ baseline total difficulties score, and household CHAOS score, children with a primary caregiver who acquired a mental health disorder had on average a 0.55 point lower increase in prosocial behavior score compared to children with a primary caregiver who had persistent absence of a mental health disorder (p = 0.021) (Table 4). Those with persistent presence or resolution of a caregiver disorder did not differ significantly from those with persistent absence of a caregiver disorder with respect to change in child prosocial behavior over the two-year time frame.

**Table 4:** Association between change in presence of a primary caregiver mental health disorder† and change in child prosocial behavior score between wave 1 and 2.

		*β*	95% CI		p-value
**Unadjusted association**	Persistent Presence	-0.18	-0.58	0.23	0.3922
	Resolution	0.08	-0.22	0.39	0.5951
	Acquisition	-0.09	-0.50	0.32	0.6581
**Adjusted association ‡**	Persistent Presence	-0.36	-0.94	0.23	0.2330
	Resolution	-0.09	-0.39	0.22	0.5704
	Acquisition	-0.55	-1.02	-0.08	0.0210*

Linear GEE regression [13] treating using reference of persistent absence of a mental health disorder

**†**Although “disorder” is used, the CDQ is a screening tool and not a clinical assessment resulting in a definitive diagnosis

**‡**Adjusting for early child-care, child HIV status, child SDQ baseline prosocial score, child SDQ baseline total difficulties score, and CHAOS score

### Power analysis

For 1087 children and their primary caregivers, a post hoc power analysis reported 73.4% power to detect a change of 0.57 in a child’s prosocial score accounting for all four-categories of their primary caregiver’s mental health disorder change between wave 1 and 2, adjusting for all five covariates (*α* = 0.05).

## Discussion

In the unadjusted analysis, a significant association was not found between a change in caregiver mental health status and change in child prosocial score between wave 1 and 2. However, after adjusting for covariates, children whose primary caregiver *acquired* a mental health disorder between wave 1 and 2 had a significantly lower increase in prosocial score compared to children whose primary caregiver had persistent absence of a mental health disorder at both wave 1 and 2 (Table 4).

The adjusted analysis showed a significantly lower increase in prosocial score for children with a primary caregiver who acquired a mental health disorder. This is consistent with prior publications reporting that compromised caregiver-child relationships, such as a caregiver acquiring a mental health disorder, can negatively impact a child’s intellectual and behavioral development [33, 39, 53, 54]. However, no significant change was observed for children whose primary caregivers had a resolved a mental health disorder. This may be due to a ceiling effect, which became evident when controlling for child SDQ baseline prosocial scores. The high prosocial baseline scores in this cohort (mean = 8.50) may have limited our ability to detect a statistically significant difference at the upper end of the prosocial subscale and thus constrained our conclusions about whether or not there is a measurable change in prosocial behavior in children whose primary caregiver resolved a mental health disorder between wave 1 and 2. Children with a stable (persistent presence) or positive change (resolution) in their caregiver’s mental health disorder status continued on the same trajectory as those in the reference group, those whose caregivers never had a mental health disorder (persistent absence). In contrast, children with a negative change (acquisition) in their caregiver’s mental health disorder status continued to increase in prosocial score but at a significantly reduced trajectory (p = 0.021) (Table 4). Since wave 1 adjusted cross-sectional analysis showed no statistical association between child prosocial score and caregiver mental health disorder at baseline (Table 2), these data suggest that a caregiver acquiring a mental health disorder during the time period studied had an independent and significant effect on a child’s subsequent prosocial development.

Our findings suggest that changes in caregiver mental health may have a smaller than expected effect on changes in child prosocial behavior in this population (Table 1). This may be in part due to the ceiling effect described above. However, this analysis covered a time frame of only two years and prosocial development may be ongoing. If the acquisition of a mental health disorder has a sustained impact on child prosocial behavior, it is possible that a larger and more clinically significant difference in prosocial behavioral development might be observed over a longer time period, particularly as the child transitions into adolescence and adulthood. The possibility of a cumulative effect over a longer time period warrants further investigation, and may be possible using the recently completed Asenze Cohort Study’s wave 3 that collected both CDQ data on primary caregivers and SDQ data on adolescents at an average age of 16.5 years.

The study has a number of limitations. First, although the sensitivity and specificity of the CDQ were within the acceptable range for a community screen and the validity was tested in the study population during wave 2, the CDQ is a screening tool and does not provide a definitive clinical diagnosis [30]. As such, it is designed to place study subjects in a high-risk or low-risk group and cannot be relied upon to definitively detect the presence or absence of a mental health disorder nor differentiate between various psychiatric disorders [30]. Moreover, not all mental health disorders are alike. Different disorders may have distinctly different effects on child prosocial development that were not captured in these analyses as there was insufficient sample size and therefore inadequate power to sub-analyze the effect of specific mental health disorders on child prosocial development. Further studies will be needed to investigate the effects of each of the five mental health disorders on child prosocial development. Second, the SDQ prosocial subscale reported a marginally acceptable internal consistency, which may have impacted our ability to accurately measure changes in child prosocial behavior [29]. Third, the increase in child prosocial behavior during the two-year interval as measured by SDQ may represent normal increases in prosocial behavior as children develop cognitively and emotionally with age [1, 34]. Moreover, the fact that only two time periods were included in the analysis restricted our ability to observe actual trends between or beyond these two time points, leading us to assume that the relationship was roughly linear over time. Fourth, the information on child prosocial behavior as reported by primary caregivers along with published data suggest that a caregiver’s perception of their child’s prosocial behavior may not reflect the child’s true behavior and that factors that impact a caregiver’s psychological functioning [29], such as the presence of a mental health disorder, may further skew this perception [32]. This may result in an inaccurate representation of the true prosocial development of the children under study. One study on a cohort of heterosexual couples and their elementary school aged children in Milan, Italy, found that parental perception of their child’s behavior varied according to their stress level [55]. Another study of children aged 3 to 6 years old in Italy noted that parents reported higher levels of child prosocial behavior compared the same child’s teacher [56]. Since the Asenze Cohort Study measures SDQ prosocial scores of teachers at wave 2, we propose a follow-up sensitivity analysis comparing prosocial behavior scores from a teacher’s response to that from the primary caregiver at wave 2 [35].

This study has a number of strengths. First, existing literature on prosocial behavior is almost exclusively cross-sectional in nature. Our longitudinal study design allowed for the establishment of a clear temporal association. By using a positive change in health outcome over time, we were able to establish a significant unidirectional relationship between exposure and outcome. Stated differently, since all children would be expected to show an increase in prosocial development over time, our study design allowed us to measure and reach meaningful conclusions about relative not absolute increases in prosocial score. Second, the CDQ and SDQ are widely used measures whose internal validity have been assessed in the population under study and whose construct validity have been upheld through comparisons with other widely accepted measures [29, 30]. Third, since a child’s behavioral development is impacted and perceived differently by different cultures, it is important that research that informs KwaZulu-Natal and South African policies and interventions come from within this population, including findings from this study [29]. By analogy, a study of families in Finland whose parents had a mental health condition found that the implementation of culturally-relevant discussion-based interventions improved the children’s prosocial behavior [32]. This highlights the importance of providing child-level and culturally-relevant interventions in the presence of parental mental health conditions to promote children’s psychosocial development. This is perhaps best illustrated by the ongoing Khulakahle Mntwana Program supported by The Valley Trust. This interventional program, based on the findings of waves 1 and 2 of the Asenze Cohort Study, uses group counseling sessions and home visits to support the KwaZulu-Natal population, including caregivers who experience psychological distress. This paper’s conclusions about the importance of caregiver mental health as a factor impacting child prosocial development provides continued evidence in support of this program. Lastly, a power of 73.4% boosts confidence in the validity of our findings.

## Conclusions

These findings provide support for the hypothesis that a child’s prosocial behavioral development over time may be affected by changes in the acquisition of a mental health disorder in their primary caregiver. Continuing to follow this cohort through adolescence and early adulthood will provide an even better understanding of this association. This study provides opportunity to develop effective interventions to promote prosocial development in children and adds force to the need for effective mental health disorder prevention and treatment, especially for those caring for children. It is also important that any intervention aimed to positively impact childhood prosocial development be grounded in contextually-relevant in-country studies that take into account the unique history and culture of the community.
